# Consensus formalisé: recommandations de pratiques cliniques pour la prise en charge de la migraine du patient adulte africain

**DOI:** 10.11604/pamj.2016.24.81.8695

**Published:** 2016-05-25

**Authors:** Mahmoud Ait Kaci Ahmed, Monia Haddad, Beugré Kouassi, Hamid Ouhabi, Alain Serrie

**Affiliations:** 1 Hôpital Ait Idir, Alger, Algérie; 2 Hôpital La Rabta, Tunis, Tunisie; 3 Centre Hospitalier Universitaire de Cocody, Abidjan, Côte d'Ivoire; 4 Hôpital Militaire d'Instruction Mohammed V, Rabat, Maroc; 5 Hôpital Lariboisière de Paris, France

**Keywords:** Migraine, Afrique, recommandation, Migraine, Africa, recommandation

## Abstract

La migraine est une céphalée primaire (selon les derniers critères de l'International Headache Society) qui affecte environ 8% de la population africaine. Les femmes sont plus fréquemment touchées que les hommes et les crises apparaissent le plus souvent avant l’âge de 40 ans. Bien qu'un certain nombre de traitements, de mesure hygiéno-diététiques, et d'autres méthodes non pharmacologiques permettent de limiter l'intensité et la fréquence des crises, la prise en charge médicamenteuse de la crise de migraine est très souvent nécessaire. La disponibilité des traitements et l'accès aux soins diffèrent sur le continent africain et ont conduit à la réalisation du 1^er^ consensus d'experts pour la prise en charge du patient adulte africain. Destiné aux praticiens, ce travail collaboratif multinational a pour objectif de fournir 16 recommandations de pratiques cliniques simples, fondées sur les preuves, et adaptées aux conditions de l'exercice médical en Afrique.

## Abstract (en)

Migraine is a primary headache disorder (according to the latest International Headache Society criteria) affecting approximately 8% of African population. Women are more often affected than men and attacks usually occur before the age of 40 years Although some treatments, hygienic-dietary measures and other non-pharmacological methods can reduce the intensity and frequency of attacks, medicinal treatment of migraine attack is often necessary. Availability of treatments and access to care differ in Africa and led to the implementation of the first expert consensus recommendations for the management of the migraine in african adult patients. This multinational collaborative study is intended for health practitioners. It aims to provide 16 simple, evidence-based recommendations and is adapted to african medical practice.

## Introduction

La migraine est une céphalée primaire, survenant par crises et pouvant se manifester avec et sans aura. L'aura correspond à un dysfonctionnement neurologique focal et transitoire qui précède la céphalée dans 93% des cas. L'aura visuelle est la plus fréquente (scotome scintillant et phosphènes), mais chez certains patients elle peut également être sensitive (fourmillements ou engourdissements unilatéraux) ou aphasique (trouble transitoire du langage) [1]. La migraine sans aura, autrefois appelée migraine commune ou hémicrânie simple, est la forme la plus fréquente (69 à 79% des cas) (Tableau 1). La migraine avec aura, ou migraine classique, est retrouvée chez 10 à 33% des patients migraineux (Tableau 2). D'un point de vue méthodologique, les études épidémiologiques sur la migraine sont difficiles à réaliser et les résultats sont parfois discordants. En effet, l'absence d'une définition standard, l'hétérogénéité de la maladie, parfois chez le même individu (intensité, fréquence, durée, symptômes associés,…), le phénomène de télescopage (fait de rapporter dans le présent des évènements survenus dans le passé) représentent autant de biais et freinent la réalisation d’études prospectives [2, 3]. La classification des céphalées de l'International Headache Society (IHS) de 2004 a cependant permis d'adopter un langage commun, d’éviter le biais de la définition de la migraine et de limiter les variations des résultats entre les études épidémiologiques [4]. Il faut également ajouter que certains patients ne consultent pas, ou alors de manière retardée par rapport à l'apparition des symptômes [2]. Une large étude réalisée aux Etats-Unis a pu réduire les biais méthodologiques par une méthode mathématique et a estimé, à partir d'appels téléphoniques, l'incidence de la migraine selon l’âge et le sexe durant l'année 1986-1987 [3]. D'après les auteurs, l'incidence annuelle de la maladie est de 137/100 000 hommes et de 294/100.000 femmes. Les résultats indiquent que la migraine avec aura commence 3 à 5 ans plus tôt que la migraine sans aura chez les deux sexes, et que la maladie commence plus tard chez les femmes par rapport aux hommes. Avant l’âge de 12 ans, la prévalence de la maladie est identique dans les deux sexes ou légèrement plus élevée chez les garçons. Elle augmente ensuite après l’âge de 12 ans et prédomine chez les femmes. Dans les pays européens et en Amérique du Nord, environ 12% de la population souffrirait d’épisodes migraineux, ce chiffre chutant à 8% dans les pays en voie de développement comme la Chine ou l'Afrique noire [1, 5–13]. Dans l’étude de Stewart et al., intégrant des patients de plusieurs ethnies vivant aux Etats-Unis, les caucasiens avaient la prévalence la plus élevée (20,4% chez les femmes et 8,6% chez les hommes). Les afro-américains avaient un taux de prévalence de 9,2% chez les femmes et de 4,8% chez les hommes. Ces résultats suggèrent la présence d'une susceptibilité génétique à la migraine [2, 14]. La migraine est une maladie significativement invalidante. Elle limite l'activité quotidienne de plus de 50% des patients et empêche toute activité dans 25 à 30% des cas [2]. Les répercussions de la maladie migraineuse sur le travail et la scolarité sont donc très fréquentes. Selon les études, le nombre annuel moyen de jours d'absentéisme chez les migraineux varie entre 1,4 et 4 jours [15]. Mais même si certains patients ne s'absentent pas durant leurs crises, leur efficacité dans le travail est nettement diminuée [2, 14, 16–18]. Des problèmes sociaux extra et intrafamiliaux sont également souvent recensés [15, 19].

**Tableau 1:** Migraine sans aura, critères diagnostiques selon la classification de l'IHS 2004

Critère	Description
A	Au moins cinq crises répondant aux critères B, C ou D
B	Crises de céphalées durant de 4 à 72 heures (sans traitement)
C	Céphalées ayant au moins deux des caractéristiques suivantes:
	unilatérale
	pulsatile
	modérée ou sévère
	aggravée par les activités physiques de routine, telles que montée ou descente des escaliers
D	Associé aux céphalées, au moins l'un des symptômes suivants:
	nausées et/ou vomissements
	photophobie et phonophobie
E	Au moins un des éléments suivants:
	l'histoire, l'examen physique et neurologique ne suggèrent pas de désordre organique
	l'histoire, l'examen physique et neurologie suggèrent un désordre organique mais celui-ci est écarté par la neuro-imagerie ou tout autre procédé de laboratoire
	un désordre organique existe mais les crises migraineuses ne sont pas apparues pour la première fois en liaison temporelle directe avec ce désordre

**Tableau 2:** Migraine avec aura, critères diagnostiques selon la classification de l'IHS 2004

Critère	Description
A	Au moins deux crises répondant aux critères B
B	Aura ayant une des caractéristiques suivantes sans signe moteur: Symptômes visuels totalement réversibles soit positifs: scotomes lumineux, scintillements… ou négatifs: amaurose transitoire partielle ou totale Symptômes sensoriels totalement réversibles soit en plus: fourmillements, brûlures, soit en moins paresthésies engourdissements aphasie ou autres troubles du langage transitoires et totalement réversibles
C	Au moins deux des caractéristiques suivantes: signes visuels homonymes le symptôme de l'aura se développe progressivement sur plus de cinq minutes et en cas de deux ou plusieurs symptômes, ils surviennent successivement la durée de chacun des symptômes de l'aura n'excède pas 60 minutes. S'il y a plusieurs symptômes, la durée acceptée est augmentée en conséquence.
D	La céphalée qui a les caractéristiques de la migraine sans aura, fait suite à l'aura après un intervalle libre de moins de 60 minutes, mais peut parfois commencer avant l'aura ou lui être contemporaine.
E	Il n'y a pas d'autre étiologie pouvant expliquer ce phénomène

## Méthodes

Ces recommandations ont été élaborées par la méthode du consensus formalisé, de type Delphi-modifié, décrite par la Haute Autorité de Santé (HAS) française (HAS, 2010). Elle a reposé, d'une part, sur l'analyse et la synthèse critiques de la littérature médicale disponible (PubMed), et, d'autre part, sur l'avis et les données non publiées d'un groupe de 16 spécialistes pratiquant sur le continent africain qui a réalisé une cotation en deux tours des propositions de recommandations établies par le groupe de pilotage.

**Membres du comité de pilotage**

Pr Mahmoud AIT KACI AHMED, Chef de service de neurologie, Hôpital Ait Idir, Alger (Algérie)

Pr Monia HADDAD, Centre de Traitement de la douleur, Hôpital La Rabta, Tunis (Tunisie)

Pr. Beugré KOUASSI, Neurologue, Université FHB, CHU de Cocody, Abidjan (Côte d'Ivoire)

Pr Hamid OUHABI, chef de service de neurophysiologie, Hôpital Militaire d'instruction Mohammed V, Rabat (Maroc)

Pr Alain SERRIE, Professeur associé des universités, Chef de service, Hôpital Lariboisière de Paris, (France)

**Membres du comité de relecture et de cotation**

Dr Patrice BARASUKANA, Professeur à l'Université du Burundi, Faculté de médecine de Bujumbura (Burundi)

Pr Lahoucine BARROU, Enseignant à la faculté de médecine et pharmacie de Casablanca, Directeur du Diplôme Universitaire d'Evaluation et Traitement de la Douleur, Chef de service d'anesthésie réanimation au CHU Ibn Rochd Casablanca, Président du Comité de lutte contre la douleur (CLUD), Casablanca (Maroc)

Pr Mofou BELO, Chef du service de neurologie, CHU Sylvanus OLYMPIO, Lomé (Togo)

Dr Noureddine BENGAMRA, Neurologue, ancien chef de service de CHU, Directeur de clinique de Neurologie, Oran (Algérie)

Pr Ali CHERIF, Chef de service d'Anesthésie-Réanimation, Président de jury d'examens, Directeur du centre Tunisie de la Comité de l'European society of Anesthesiology (CEEA), Président du comité scientifique de la Société Tunisienne d'Anesthésie-Réanimation, CHU La Rabta Tunis, Faculté de Médecine de Tunis (Tunisie)

Dr Benjamin CLET TCHALEU NGUENKAM, Neurologue Enseignant, Hôpital général de douala Université des Montagnes, (Cameroun)

Pr Smail DAOUDI, Responsable du département de neurologie à la faculté de médecine Mouloud Maameri, médecin chef du service de neurologie, responsable de l'unité douleur CHU Nedir Mohamed de Tiziouzou (Algérie)

Pr Catherine DZIRI, Professeur en Médecine Physique-Réadaptation Fonctionnelle à la Faculté de Médecine de Tunis et chef de service à l'Institut National d'Orthopédie M. Kassab, Université de Tunis El Manar, (Tunisie)

Pr Rachid HSSAIDA, Anesthésiste réanimateur, Spécialiste dans le traitement de la douleur, Hypnose clinique et acupuncture Hôpital Militaire Rabat (Maroc)

Pr Jean Pierre ILUNGA MWENA, Chef des Travaux au Département d'anesthésie et Réanimation des Cliniques Universitaires de Kinshasa, à l'université de Kinshasa (RDC Congo)

Pr B Jean KABORE, Chef du service de Neurologie du CHUYO de Ouagadougou (Burkina Faso)

Pr Moustapha NDIAYE, Neuropédiatre, CHU Fann Dakar (Sénégal)

## Etat actuel des connaissances

### Aspects cliniques et démarche diagnostique

**Recommandation 1 - accord professionnel fort:** Dans le contexte africain, il n'existe pas d'arguments pour définir des critères diagnostiques de la migraine différents de ceux proposés par l'IHS. Le tableau clinique de la crise migraineuse varie selon les individus [4]. Dans 40% des cas, la crise est précédée de prodromes. Ces prodromes sont à type d'irritabilité, d'asthénie, de somnolence, d'une tendance dépressive, d'euphorie, d'une sensation de faim ou de constipation [1]. La crise peut commencer à n'importe quel moment de la journée et durer entre 4 et 24 heures [20, 21]. Son intensité est difficile à standardiser, mais les études utilisant les critères de l'IHS révèlent qu'elle est sévère chez 75 à 84% des patients [1]. La céphalée est souvent unilatérale et pulsatile et accompagnée d'autres symptômes: nausées (91% des cas), photophobie (77% des cas), vomissements (50% des cas). On retrouve plus rarement: une obstruction nasale ou une rhinorrhée, une constipation ou une diarrhée, un larmoiement, une somnolence, une irritabilité, un état dépressif et des troubles de la concentration. La fréquence des crises est variable d'un patient à l'autre et chez le même individu, bien que les femmes aient des crises plus fréquentes que les hommes [15]. Plus de 56% des patients signalent une amélioration des céphalées par le sommeil (repos de 45 minutes à 4 heures) [16]. Le diagnostic positif de la migraine repose sur l'interrogatoire, qui doit être complété par un examen clinique complet. L’évolution par crises entre lesquelles le patient ne souffre pas est un élément essentiel au diagnostic. En 2004 l'International Headache Society (IHS) a publié une nouvelle classification des céphalées permettant ainsi de standardiser la nomenclature et de fournir des critères diagnostiques plus précis (Tableau 1 et Tableau 2) [4].

**Recommandation 2 - accord professionnel fort:** Le diagnostic de migraine est clinique. Aucun examen complémentaire biologique ou radiologique n'est recommandé. Dans les formes typiques de migraine, les examens complémentaires sont inutiles. Ils sont indiqués lorsque le tableau clinique est atypique (en cas de migraine compliquée ou d'anomalies à l'examen neurologique) et dans le cadre de travaux de recherche [4]. L'IRM cérébrale est le plus souvent normale au cours de la migraine, mais des anomalies de la substance blanche sont retrouvéesdans 12 à 39,6% des cas [22–24].

**Recommandation 3 - accord professionnel fort:** Compte-tenu de la fréquence des comorbidités cardio-vasculaires, une évaluation est recommandée, selon le contexte, pour la prise en charge du patient migraineux.

Plusieurs études ont été réalisées dans le but de préciser la relation entre la migraine et d'autres maladiesconcomitantes. Merikangas et Fenton ont en effet mis en évidence une augmentation du risque d'infarctus du myocarde (x 1,8) et du risque d'angine de poitrine (x 1,9) chez les patients migraineux par rapport à la population générale [25]. De plus, de nombreuses études révèlent que la migraine est un facteur de risque d'accident vasculaire cérébral (AVC) ischémique, notamment chez les femmes jeunes (risque relatif 2,8-4,3) ayant une migraine avec aura (risque relatif6,2-8,6). Cette association est encore plus importante en présence d'autres facteurs de risque comme le tabac (x 10,2) et les contraceptifs oraux (x 13,9). Dans plus de 40% des cas, l'AVC s'installe au décours immédiat d'une crise de migraine (infarctus migraineux). Selon Welchet al, l'infarctus migraineux représente 25% des infarctus cérébraux [26].

### Prise en charge médicamenteuse de la migraine

#### Traitement de la crise migraineuse

**Recommandation 4 - accord professionnel fort:** Chez l'adulte et l'enfant (> 6 ans), la prescription d'un AINS est recommandée en 1^ère^ intention. L'aspirine et le paracétamol sont des alternatives possibles. Eventuellement, l'association au métoclopramide peut être envisagée en cas de nausées importantes et en l'absence de contre-indications.

**Recommandation 5 - accord professionnel fort:** Compte-tenu de leur faible disponibilité et de leur coût dans le contexte africain, le recours à un triptan est généralement recommandé comme une stratégie thérapeutique de 2^ème^ intention.

**Recommandation 6 - accord professionnel fort:** Il est recommandé de prendre le traitement précocement, dès l'apparition de la crise pour les antalgiques de palier I, et dès l'apparition de la douleur pour les triptans.

Le traitement des crises de migraine dépend de leur fréquence, de leur sévérité et de la symptomatologie. Il existe deux types de traitements [27]: les traitements non spécifiques (antalgiques et anti- inflammatoires non stéroïdiens): les anti-inflammatoires non stéroïdiens (AINS): le naproxène, l'ibuprofène, le kétoprofène et le diclofénac (grade A); l'aspirine en monothérapie (grade A), éventuellement associée au me'toclopramide (grade A) pour améliorer les troubles digestifs (accord professionnel); le paracétamol en monothérapie (grade C). L'association de la caféine au paracétamol et à l'aspirine n'a pas fait la preuve clinique d'une potentialisation d'effet et ne peut pas être recommandée, d'autant qu'il n'est pas exclu que la caféine induise un abus médicamenteux, voire un comportement addictif (accord professionnel). Il est recommandé d’éviter les opioïdes (codéine, tramadol, morphine et autres opioïdes forts), seuls ou en association, qui peuvent aboutir à un abus médicamenteux, voire à un comportement addictif (accord professionnel).

**les traitements spécifiques** (triptans et dérivés ergotés) qui agissent sur les récepteurs 5 HT1B/D pour inhiber l'inflammation neurogène et la vasodilatation, supposées être à l'origine de la céphalée migraineuse: les triptans (grade A). L'efficacité porte sur la céphalée mais aussi sur les symptômes digestifs associés et la phono/photophobie (grade A); le tartrate d'ergotamine (grade B); la dihydroergotamine par voie per-nasale (grade A) ou injectable (grade B). Les différences d'efficacité et de tolérance entre les différentstriptanssont minimes (grade B). Un patient non répondeur à un triptan lors de la 1ère crise peut être répondeur pendant les crises suivantes (grade A).

#### Traitement prophylactique

**Recommandation 7 - accord professionnel fort:** Il est recommandé de comptabiliser le nombre total de prises de traitement par mois (agenda) afin de repérer un défaut d'observance thérapeutique ou une utilisation abusive susceptible d'entrainer des céphalées chroniques.

**Recommandation 8 - accord professionnel fort:** Une fréquence de crises supérieure à 2/semaine pendant 3 mois doit faire envisager un traitement prophylactique. La décision de mettre en route un traitement prophylactique se prend en accord avec le patient, en fonction du handicap lié à la migraine et de l'efficacité des traitements des crises. Le traitement prophylactique doit associer des mesures médicamenteuses et non médicamenteuses.

Les traitements prophylactiques de la migraine ont pour objectif principal la réduction de la fréquence des crises. Ils peuvent également apporter d'autres bénéfices pour le patient à savoir une diminution de la sévérité des céphalées, une meilleure réponse aux traitements des crises, une moindre sensibilité aux facteurs déclenchants et une meilleure qualité de vie [28, 29]. Un traitement prophylactique doit être instauré à chaque fois que [27, 30–32]: la fréquence et/ou l'intensité des crises constituent un handicap familial, social ou professionnel pour le patient; le patient consomme, depuis 3 mois, le ou les traitement(s) de crise plus de 2 jours chaque semaine, et cela même en cas d'efficacité, afin d’éviter une céphalée chronique quotidienne avec abus médicamenteux. La mise en place de cette prophylaxie doit s'associer à une démarche éducative du patient à qui il faut bien expliquer que ce traitement ne supprime pas les crises mais réduit leur fréquence et leur intensité. La tenue d'un agenda des crises permettra de mieux apprécier l'efficacité de cette prophylaxie.

**Recommandation 9 - accord professionnel fort:** En l'absence de contre-indications aux bétabloquants, le propranolol et le métoprolol sont les molécules à privilégier en 1ère intention pour le traitement prophylactique de la migraine.

Les différentes molécules utilisées dans la prophylaxie de la migraine sont classées en 3 catégories: efficacité démontrée, probable ou douteuse [27, 33]. Efficacité démontrée: certains bétabloquants (propranolol et métoprolol) et certains antiépileptiques (topiramate et valproate de sodium). Efficacité probable: amitriptyline, venlafaxine, aténolol, nadolol, timolol, nébivolol, candésartan, flunarizine, méthysergide, naproxène sodique, oxétorone et pizotifène. Efficacité douteuse: dihydroergotamine, indoramine et gabapentine. En considérant le niveau de preuves d'efficacité, la balance bénéfice/risque, l'existence d'une AMM et la disponibilité des médicaments dans nos pays, les molécules à privilégier en première intention sont le propranolol et le métoprolol, en l'absence de contre-indication à l'utilisation des bêtabloquants [34–36]. En cas de contre-indication, d'intolérance ou d'inefficacité, le choix de la molécule repose sur le terrain, les comorbidités et la sévérité de la migraine en considérant toujours la balance bénéfice/risque (poids, sédation, asthénie et risque tératogène) et l'existence d'une AMM [27, 37]. Quelques situations cliniques permettent d'orienter le choix de la molécule à utiliser [38–40]: tendance à la prise de poids: éviter l'amitriptyline, le pizotifène et le valproate de sodium, et préconiser le topiramate; syndrome de raynaud, asthme, troubles sexuels, pratique du sport: éviter les bétabloquants; tendance à la constipation: éviter l'amitriptyline; tendance dépressive, troubles du sommeil, céphalées de tension associées: préférer l'amitriptyline; hypertension artérielle associée, stress: privilégier les bétabloquants; migraine avec aura: éviter les bétabloquants et privilégier les antiépileptiques.

**Recommandation 10 - accord professionnel fort:** Le traitement prophylactique de la migraine doit être débuté en monothérapie à posologie croissante, évalué à 3 mois, et poursuivi 6 à 12 mois. En cas d’évolution favorable, il peut être interrompu progressivement. Si la fréquence des crises augmente, le traitement sera repris. En cas d’échec (réduction des crises < 50%), une association à dose minimale efficace est envisageable, en évitant la même classe thérapeutique et l'association d'un triptan à un autre vasoconstricteur. Il est recommandé de débuter le traitement prophylactique par une monothérapie à faible dose [28, 37, 38]. La posologie peut ensuite être augmentée de façon progressive pour atteindre un effet optimal. En cas de bonne tolérance, le traitement doit être poursuivi pendant 2 à 3 mois, en demandant au patient de tenir un calendrier de ses crises afin de d'apprécier son efficacité de façon objective. Le traitement de fond est jugé efficace lorsqu'il réduit la fréquence des crises d'au moins 50% [27, 34]. Il est important de tenir compte également de la diminution de l'intensité et de la durée des crises, et de la réduction de la prise de médicaments. Le traitement à dose efficace sera poursuivi pendant 6 mois à 1an, adapté aussi étroitement que possible à l’évolution spontanée de la migraine puis diminué très lentement avant d’être arrêté. Le même traitement pourra être repris si la fréquence des crises augmente à nouveau. En cas d’échec, deux possibilités peuvent être envisagées: augmentation de la posologie, en l'absence d'effets indésirables; proposition d'un autre traitement de fond. L'association de deux traitements de fond à plus faible dose peut être envisagée dans le but de réduire les effets indésirables respectifs de chaque molécule, après les avoir testés séparément. L'association d'amitriptyline avec un bétabloquant par exemple peut avoir un intérêt en seconde intention [41–43]. En cas d’échecs répétitifs, il faut évaluer l'observance ou rechercher un abus médicamenteux.

**Recommandation 11 - Accord professionnel fort:** La prise en charge de la migraine cataméniale et des migraines contemporaines de modifications hormonales de la femme (ménopause, grossesse) peut, en l'absence d'une amélioration avec le traitement usuel, nécessiter un traitement hormonal spécifique prescrit par un spécialiste. Si le traitement de la crise d'une patiente souffrant de migraine cataméniale n'est pas efficace, un traitement préventif limité à la période menstruelle peut être proposé [27, 28, 44, 45]: l'estradiol cutané (1,5 mg/jour pendant 7 jours) en débutant le 2^ème^ jour précédant la période menstruelle; le zolmitriptan (2,5 mg deux fois par jour), le frovatriptan (2,5 mg deux fois par jour) ou le naratriptan (1 mg deux fois par jour), s'ils sont disponibles et bien tolérés. Chez les patientes ayant une contraception orale, la survenue des crises menstruelles peut être prévenue grâce à la prescription d'un estroprogestatif en continu ou d'un progestatif pur [27, 44]. La migraine ne constitue pas une contre-indication au traitement hormonal de la ménopause (THM) [28]. Cependant, en cas d'aggravation des crises sous THM, notamment avec aura, on discutera un passage à une forme transdermique, une réduction des doses d'estradiol ou un arrêt du THM [45]. La grossesse étant généralement associée à une rémission partielle voire complète des crises, la mise en place d'un traitement de fond n'est pas nécessaire. En revanche, si un traitement prophylactique doit être administré, il faut préférer un bêtabloquant (propranolol ou métoprolol), ou en deuxième intention un antidépresseur tricyclique (amitriptyline) avec un suivi mensuel [33, 46, 47]. Les dérivés de l'ergot de seigle et le valproate de sodium sont formellement contre-indiqués en cas de grossesse [27, 34].

### Migraine et vie hormonale de la femme

Des liens étroits existent entre migraine et hormones ovariennes. Cette maladie pose donc des problèmes particuliers lors de certaines étapes de la vie hormonale de la femme [48]. Les crises peuvent survenir à des moments particuliers du cycle menstruel. On définit alors deux types de migraines en fonction du moment de leur survenue [49, 50]: la migraine menstruelle (24 à 56% des cas), caractérisée par des crises survenant à n'importe quel moment du cycle, mais dont la fréquence augmente au cours de la menstruation; la migraine menstruelle pure ou migraine cataméniale (7,2% des cas), lorsque les crises surviennent régulièrement et exclusivement entre 2 jours avant et 3 jours après les règles. La migraine menstruelle est probablement liée à la chute brutale du taux d’œstrogène lors de la menstruation et à une prédisposition génétique, mais le mécanisme précis n'est pas encore élucidé [50, 51]. Environ 15 à 50% des femmes rapportent une aggravation de leur maladie lors de la prise de contraceptifs oraux, et plus particulièrement durant la semaine d'arrêt de ces derniers [52]. L'association de la contraception orale avec la migraine représente un important facteur de risque d'accident ischémique cérébral (le risque est multiplié par 13,9), surtout chez les personnes fumeuses [52]. Cependant, la migraine ne représente généralement pas une contre-indication à la contraception orale, sous réserve de quelques règles de prudence [52–54]: proscrire la consommation de tabac; préférer les pilules faiblement dosées en éthynil-oestradiol ou des progestatives pures. L'amélioration de la migraine au cours de la grossesse a été démontrée dans toutes les études et concerne 60 à 70% des femmes, surtout au cours du 2^ème^ et 3^ème^ trimestre de grossesse [21, 55, 56]. Mais les crises peuvent rester inchangées chez certaines patientes et peuvent même s'aggraver, surtout durant le premier trimestre de grossesse (4 à 8%) et la semaine précédant l'accouchement [21, 55]. Dans 10% des cas, la migraine débute pendant la grossesse (migraine avec aura). Cette modification du cours évolutif de la migraine lors de la grossesse est probablement liée à la présence d'un facteur intrinsèque sensible aux œstrogènes dans les neurones hypothalamiques [50, 56, 57]. Par ailleurs, la migraine ne représente aucun facteur de risque ni pour la mère ni pour le fœtus [55, 56]. Enfin, certaines femmes rapportent une aggravation de leur migraine au cours de la pré-ménopause, principalement liée à la grande fluctuation du taux d’œstrogènes, aux troubles du sommeil et à la dépression, souvent présents au cours de cette période [9, 52, 58].

### Prise en charge non médicamenteuse de la migraine

**Recommandation 12- accord professionnel fort:** Dans le contexte africain, les traitements non médicamenteux peuvent être particulièrement bien adaptés, et éventuellement associés aux traitements médicamenteux, en respectant le contexte socio-culturel et spirituel, notamment dans les situations suivantes: préférence du patient pour les interventions non pharmacologiques; contre-indications ou mauvaise tolérance aux traitements pharmacologiques spécifiques; insuffisance ou absence de réponse aux traitements pharmacologiques; enfants, femmes enceintes ou grossesse planifiée; histoire de l'utilisation à long terme, fréquente ou excessive de médicaments analgésiques qui peut aggraver les céphalées; stress important; difficultés d'accessibilité géographique ou financières aux soins de santé

**Recommandation 13 - accord professionnel fort:** L'amélioration de l'hygiène de vie, du comportement alimentaire et l'identification des facteurs prédisposants et déclenchants modifiables, notamment à l'aide d'un agenda, sont les premières mesures recommandées lors de la prise en charge du patient migraineux. De nombreux facteurs peuvent être à l'origine du déclenchement des crises migraineuses [59, 60]: le stress (mis en cause dans 58 à 62% des cas), plusieurs aliments (chocolat (73%), fromage (48%), agrumes (30%), alcool (25%), graisses et oignons (18%), café et thé (14%)), certaines habitudes alimentaires (petit déjeuner insuffisant ou absent, repas sauté ou retardé, hypoglycémie chez un patient diabétique), des modifications du rythme de sommeil, un traumatisme crânien et/ou cervical, la lumière, l'effort physique, le changement de climat et de saison [61–64]. Les recommandations s'accordent sur le fait de passer par une phase d'identification des facteurs incriminés à l'aide d'un agenda afin de les écarter [65]. Ceci est un moyen peu coûteux de réduire les crises de migraines.

**Recommandation 14 - accord professionnel fort:** Sous réserve de leur disponibilité et de leur accessibilité financière, la relaxation et les thérapies cognitivo-comportementales sont des options thérapeutiques recommandées pour la prévention de la migraine avec un personnel formé, notamment à l’éducation thérapeutique. Elles peuvent être combinées avec une chimiothérapie préventive. Certaines méthodes, comme la relaxation, le biofeedback ou les thérapies cognitivo-comportementales, permettant de se relaxer, d’éliminer les tensions et d'apprendre à gérer le stress, ont fait leurs preuves contre la migraine (grade A) [27]. Elles peuvent également être proposées à des patients préférant le traitement comportemental, lorsque le traitement pharmacologique est contre-indiqué ou non toléré, en cas de grossesse ou de grossesse planifiée ou encore chez des patients ayant des facteurs de stress importants.

**Recommandation 15 - accord professionnel fort:** La phytothérapie a démontré son efficacité pour le traitement préventif des migraines, et peut être recommandée sous réserve de sa disponibilité locale et de l'expérience du praticien à l'utiliser.

**Recommandation 16 - accord professionnel fort:** En l'absence de niveaux de preuves suffisants, il n'est pas recommandé de recourir aux traitements fondés sur l'utilisation de l'hypnose, l'acupuncture, l'homéopathie, la stimulation électrique transcutanée, les manipulations cervicales comme thérapie préventive ou aiguë de la migraine. La prise de compléments alimentaires peut parfois éviter certaines carences, potentiellement liées aux crises migraineuses. Les substances pour lesquelles les études et preuves sont disponibles sont les suivantes [65, 66]: les petasites (butterbur) (Niveau A); le magnésium (femmes souffrant de syndrome prémenstruel, migraines avec aura) La dose généralement employée est de l'ordre de 600 mg/j. pendant trois à quatre mois. (Niveau B); la riboflavine (vitamine B2) (Niveau B); le MIG-99 (grande camomille) (Niveau B); le coenzyme Q10. La dose généralement prescrite est de 100 mg trois fois par jour. Plusieurs semaines peuvent être nécessaires avant de constater une amélioration. (Niveau C). Certaines méthodes physiques permettent de réduire le stress: le massage, la physiothérapie, la stimulation électrique transcutanée, les manipulations vertébrales et l'acupuncture [67]. Mais les résultats des études ayant évalué l'effet de ces méthodes sur le traitement de la migraine sont très controversés. Chaibi et al., dans une revue systématique des essais cliniques randomisés évaluent l'efficacité des thérapies manuelles et concluent que le massage thérapeutique, la kinésithérapie, la relaxation et la manipulation vertébrale peuvent être tout aussi efficace que le propranolol et le topiramate dans le traitement prophylactique de la migraine [68]. En revanche, les dernières recommandations françaises pour le traitement non médicamenteux de la migraine retiennent que les données de la littérature ne recommandent ni l'acupuncture ni la manipulation vertébrale (grade A) [27]. De plus, si l'acupuncture peut être disponible dans certaines villes du continent africain, son inaccessibilité géographique et financière en font une méthode marginale pour le traitement de la migraine en Afrique.

## Conclusion

La migraine est une affection qui peut toucher plus de 8% de la population africaine. Significativement invalidante, il est prioritaire d'apporter des recommandations claires, adaptées à l'exercice médical en Afrique, quant à son diagnostic et à sa prise en charge (médicamenteuse ou non).

### Etat des connaissances actuelle sur le sujet

- Les études épidémiologiques rapportent que les migraines sont fréquentes dans la population adulte;
- De nombreuses options thérapeutiques permettent de traiter les crises migraineuses.

### Contribution de notre étude à la connaissance

- Etat des lieux sur les données épidémiologiques en Afrique;
- Recommandations sur la prise en charge de la migraine, adaptées aux conditions en Afrique (en fonction de la disponibilité des traitements et de l'accès aux soins).
